# Mutant BIN1-Dynamin 2 complexes dysregulate membrane remodeling in the pathogenesis of centronuclear myopathy

**DOI:** 10.1074/jbc.RA120.015184

**Published:** 2020-11-21

**Authors:** Kenshiro Fujise, Mariko Okubo, Tadashi Abe, Hiroshi Yamada, Ichizo Nishino, Satoru Noguchi, Kohji Takei, Tetsuya Takeda

**Affiliations:** 1 Graduate School of Medicine, Dentistry and Pharmaceutical Sciences, Okayama University, Okayama, Japan; 2 National Institute of Neuroscience, National Center of Neurology and Psychiatry (NCNP), Kodaira, Tokyo, Japan; 3 Department of Pediatrics, The University of Tokyo, Tokyo, Japan

**Keywords:** dynamin, dynamin 2, BAR domain protein, BIN1, membrane remodeling, T-tubules, centronuclear myopathy, GTPase, CNM, centronuclear myopathy, GOF, gain of function, LUVs, Large unilamellar vesicles, PR, proline-rich, TLS, tubule-like structures

## Abstract

Membrane remodeling is required for dynamic cellular processes such as cell division, polarization, and motility. BAR domain proteins and dynamins are key molecules in membrane remodeling that work together for membrane deformation and fission. In striated muscles, sarcolemmal invaginations termed T-tubules are required for excitation–contraction coupling. *BIN1* and *DNM2*, which encode a BAR domain protein BIN1 and dynamin 2, respectively, have been reported to be causative genes of centronuclear myopathy (CNM), a hereditary degenerative disease of skeletal muscle, and deformation of T-tubules is often observed in the CNM patients. However, it remains unclear how BIN1 and dynamin 2 are implicated in T-tubule biogenesis and how mutations in these molecules cause CNM to develop. Here, using an *in cellulo* reconstitution assay, we demonstrate that dynamin 2 is required for stabilization of membranous structures equivalent to T-tubules. GTPase activity of wild-type dynamin 2 is suppressed through interaction with BIN1, whereas that of the disease-associated mutant dynamin 2 remains active due to lack of the BIN1-mediated regulation, thus causing aberrant membrane remodeling. Finally, we show that *in cellulo* aberrant membrane remodeling by mutant dynamin 2 variants is correlated with their enhanced membrane fission activities, and the results can explain severity of the symptoms in patients. Thus, this study provides molecular insights into dysregulated membrane remodeling triggering the pathogenesis of *DNM2*-related CNM.

Centronuclear myopathy (CNM) is a congenital myopathy characterized clinically by muscle weakness and pathologically by the presence of centralized nuclei on muscle biopsy (1). Disrupted or disorganized T-tubules or triads in the skeletal muscles are also common pathological observations in CNM tissue (2). *BIN1*, which encodes an N-terminal amphipathic helix Bin/Amphiphysin/Rvs-homology (N-BAR) domain protein BIN1 (Bridging integrator 1)/Amphiphysin 2, has been identified as one of the causative genes for this disease (3, 4, 5, 6, 7, 8, 9, 10). BIN1 generates membrane invagination and recognizes the membrane curvature (11, 12). Among 11 splicing isoforms of BIN1, isoform 8 is specifically expressed in the skeletal muscle (13) and encodes an amphipathic H0 helix, a BAR domain, a phosphoinositide (PI) domain in its N-terminus, and a Src homology 3 (SH3) domain in its C-terminus (14, 15). The C-terminal SH3 domain serves as an interacting site with proline-rich (PR) domain-containing proteins such as dynamin 2 (3, 16). To date, two recessive CNM mutations, p.Q573∗ and p.K575∗ (p.Q434∗ and p.K436∗ in isoform 8), have been identified in CNM patients, and both mutations cause truncation of the SH3 domain. In fact, the p.K436∗ mutation was shown to abolish the BIN1–dynamin 2 interaction and recruitment of dynamin 2 to the BIN1-mediated T-tubule-like structures (TLS) (3), while p.Q434∗ causes defective triad organization ranging from abnormal orientation of the striated structures to membranous aggregation in patient biopsy tissue (17).

Dynamin is a large GTPase that plays essential roles for membrane fission in endocytosis (18, 19). Dynamin contains an N-terminal G-domain, Middle domain, pleckstrin homology (PH) domain, GTPase effector (GE) domain, and a C-terminal PR domain (PRD). The G-domain is responsible for GTP hydrolysis (20) and the Middle- and GE-domains form “stalk” structure necessary for self-assembly (21). G-domain and stalk are connected *via* a flexible hinge called Bundle Signaling Element (BSE) (21). PH domain is required for binding to negatively charged phosphoinositides including PI(4,5)P_2_ (22). *DNM2* that encodes the ubiquitously expressed dynamin isoform in mammals, dynamin 2, is another causative gene for CNM (1). Dynamin 2 localizes along Z-lines in skeletal muscles (23, 24, 25) and is thought to be involved in determination of the T-tubule orientation (26) as expression of mutated dynamin 2 has been shown to induce disruption of sarcomeres in skeletal muscles of mice and zebrafish (23, 27) as well as T-tubule fragmentation in *Drosophila melanogaster* (28). These data suggest that CNM-associated mutations in *BIN1* and *DNM2* might affect membrane remodeling activities of their protein products leading to aberrant formation and/or maintenance of the T-tubules in CNM muscles. However, the exact molecular mechanism of this defective membrane remodeling arising from these genetic mutations remains to be clearly elucidated.

In this study, using an *in cellulo* reconstitution assay, we show that wild-type dynamin 2 interacts with BIN1 to stabilize the BIN1-mediated TLS in mouse myoblast C2C12 cells. GTPase activity of dynamin 2 is suppressed by interaction with BIN1 enabling its stabilizing function of the TLS. In contrast, CNM-associated mutations in dynamin 2 produce constitutive active GTPase activity due to enhanced self-assembly and impaired GTPase suppression by BIN1. Interestingly, these results are consistent with a gain of function (GOF) of mutant dynamin 2 to establish the correlation between cellulotypes and disease phenotypes. Our results suggest that aberrant regulation of membrane remodeling by BIN1-dynamin 2 complex is tightly linked to the pathogenesis of CNM.

## Results

### Dynamin 2 stabilizes TLS reconstituted by BIN1 overexpression

**Figure 1:**
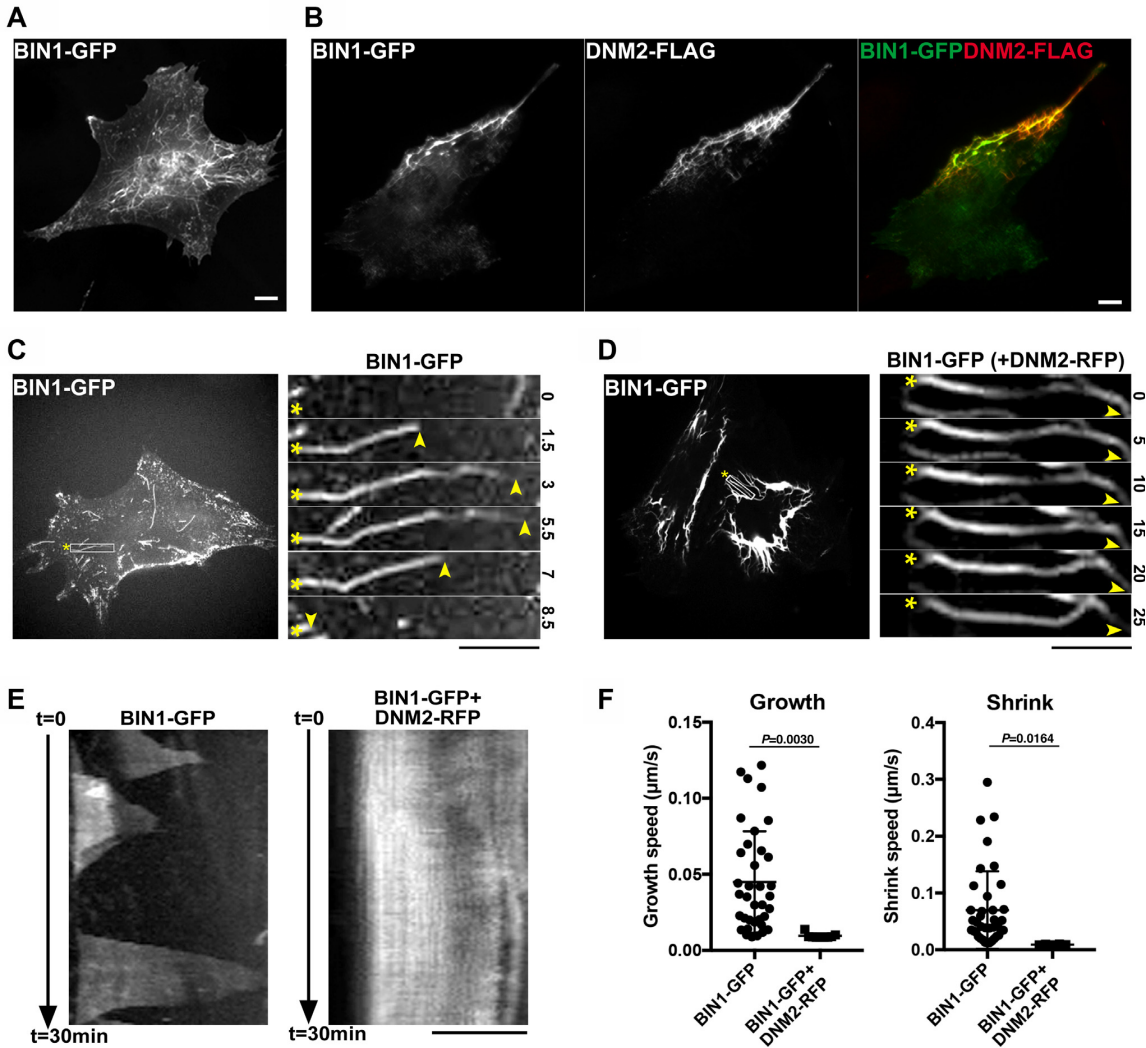
**Dynamin 2 stabilizes the BIN1-mediated TLS.***A*, TLS induced by overexpression of human BIN1 in C2C12 cells (BIN1-GFP). *B*, BIN1-mediated TLS in the presence of dynamin 2. Localization of human BIN1 (BIN1-GFP), human dynamin 2 (DNM2-FLAG) and their merged images are shown. *C*, time-lapse images of a TLS formed by sole BIN1 (*rectangle*) at different time points (0, 1.5, 3, 5.5, 7, and 8.5 min) from live imaging data (Movie S1). Proximal (*asterisks*) and distal ends (*arrowheads*) of the membrane tubule are indicated. *D*, time-lapse images of a TLS in the presence of dynamin 2 (*rectangle*) at different time points (0, 5, 10, 15, 20, and 25 min) from live imaging data (Movie S2). Proximal (*asterisks*) and distal ends (*arrowheads*) of the membrane tubule are indicated. *E*, kymograph of the TLS formed by BIN1 alone (BIN1-GFP) or BIN1 and dynamin 2 (BIN1-GFP + DNM2-RFP). *F*, scatter plot of growth and shrink speed of the TLS formed by BIN1 alone (BIN1-GFP) or in the presence of dynamin 2 (BIN1-GFP + DNM2-RFP). Average speed of growth and shrink of TLS formed by BIN1 alone are 0.04 ± 0.03 μm/s (n = 36) and 0.07 ± 0.07 μm/s (n = 38) and those formed with dynamin 2 are 0.01 ± 0.002 μm/s (n = 9) and 0.01 ± 0.001 μm/s (n = 8), respectively. Data are means ± SD. Scale bars are 10 μm.

See Figures 1 through 5. BIN1 interacts with dynamin 2 and their cooperative function is implicated in T-tubule formation (3, 14), yet the precise role of dynamin 2 was unclear. To examine dynamin 2 function in T-tubule formation, we analyzed the effect of dynamin 2 overexpression using *in cellulo* reconstitution assay for TLS (3, 7, 14, 29). Consistent with previous studies (3, 7, 14, 29), BIN1 overexpression in C2C12 cells induced numerous TLS, on which BIN1 was highly concentrated (Fig. 1*A*), whereas overexpressed dynamin 2 was found to be evenly distributed in cytoplasm of C2C12 cells (Fig. 4*B*, DNM2WT-FLAG). In contrast, when BIN1 and dynamin 2 were coexpressed, dynamin 2 was recruited to the BIN1-mediated TLS, and thick and unevenly distributed membrane tubules were induced (Fig. 1*B*). Consistent with previous studies (14, 29), these TLS appeared to be membranous structures that could be counter stained with a fluorescent membrane dye DiO (Fig. S1*A*). We also examined the membrane tubules reconstituted *in vitro* by electron microscopy. Purified BIN1 alone (Fig. S1*B*, BIN1 WT) or in combination with purified dynamin 2 (Fig. S1*B*, Dynamin 2 WT) resulted in formation of similar membrane-associated tubular structures (Fig. S1*C*).

We next examined whether BIN1 and BIN1+Dynamin tubules have similar dynamic properties. We observed TLS formed by sole BIN1 overexpression to be highly dynamic (Fig. 1*C*; Movie S1), whereas those with dynamin 2 were static (Fig. 1*D*; Movie S2). Kymograph analyses showed that the TLS formed by BIN1 alone repeatedly grew and shrank with average speed of 0.04 μm/s (n = 36) and 0.07 μm/s (n = 38), respectively (Fig. 1, *E*–*F*, BIN1-GFP). In contrast, the TLS almost stalled (growth: 0.01 μm/s (n = 9) and shrink: 0.01 μm/s (n = 8)) when dynamin 2 is coexpressed (Fig. 1, *E*–*F*, BIN1-GFP + DNM2-RFP). These results suggest that dynamin 2 might play a novel role in stabilizing TLS arising from BIN1 overexpression.

### Interaction between dynamin 2 and BIN1 is required for stable TLS formation

**Figure 2:**
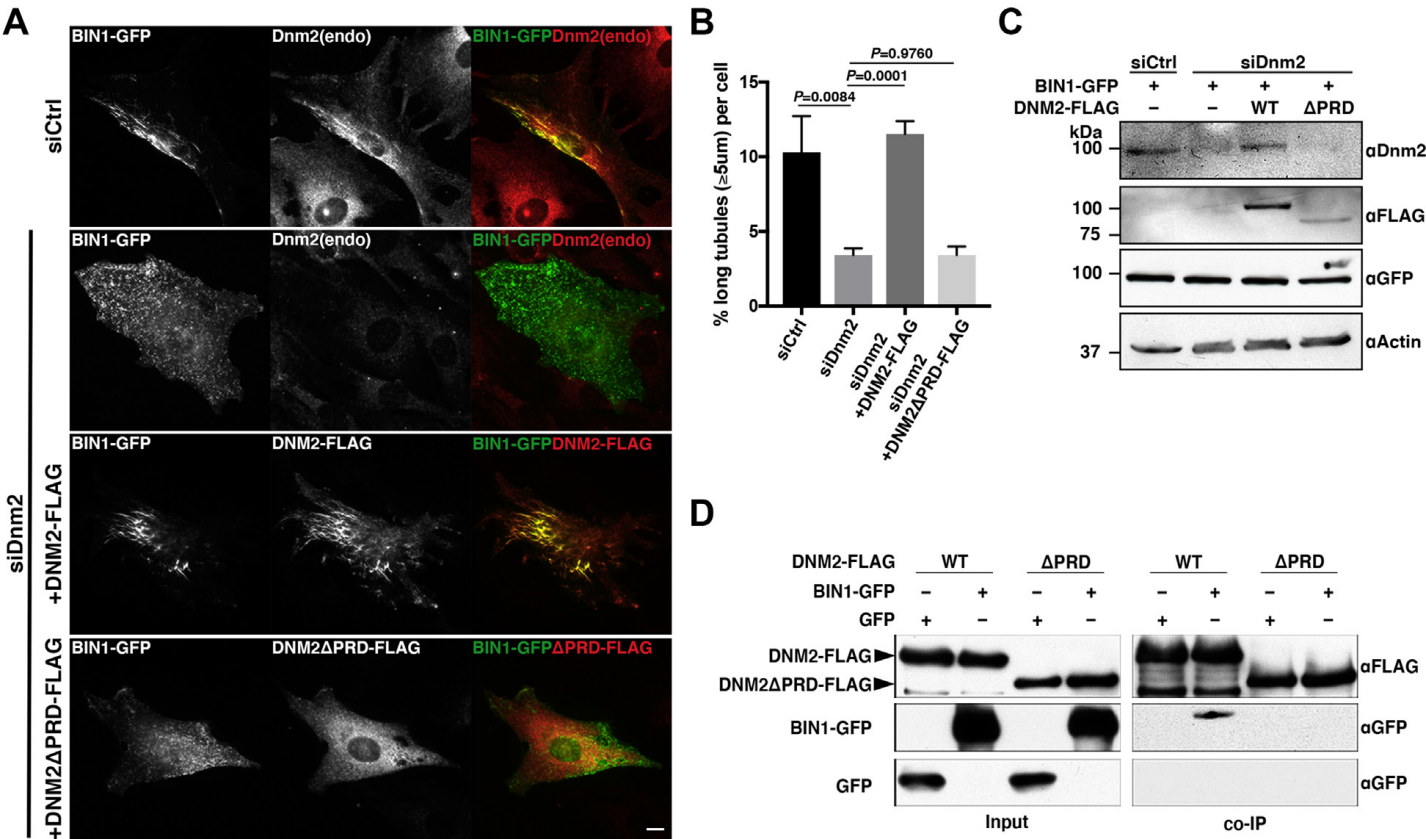
**Dynamin 2 is required for formation of the TLS.***A*, depletion of dynamin 2 inhibits TLS formation. Localization of BIN1 (BIN1-GFP), endogenous dynamin 2 (Dnm2(endo)) and their merged images in C2C12 cells treated with control siRNA (siCtrl) or dynamin 2 siRNA (siDnm2) with either exogenously expressed full-length (+DNM2-FLAG) or PRD truncated (+DNM2ΔPRD-FLAG) human dynamin 2. Scale bar is 10 μm. *B*, quantification of TLS formation in the RNAi and rescue experiment. Proportion of TLS with 5 μm or longer in length was 10.3 ± 2.4% for control RNAi cells (siCtrl), 3.4 ± 0.4% for Dnm2 RNAi cells (siDnm2), 11.5 ± 0.9% for Dnm2 RNAi cells exogenously expressing full-length human dynamin 2 (siDnm2 + DNM2-FLAG) and 3.4 ± 0.6% for Dnm2 RNAi cells with PRD truncated human dynamin 2 (siDnm2 + DNM2ΔPRD-FLAG). Data are means ± SD (n > 1600 TLS, N = 3). *C*, expression profile of endogenous and exogenously expressed dynamin 2 in the RNAi-rescue experiment. Immunoblot analysis using anti-dynamin 2 (αDnm2) and anti-FLAG (αFLAG), anti-GFP (αGFP), and anti-actin (α-Actin) are shown. *D*, dynamin 2 PRD is required for binding to BIN1. Coimmunoprecipitation analysis of full-length (WT) or PRD truncated (ΔPRD) dynamin 2 with either BIN1 (BIN1-GFP) or negative control (GFP). Immunoblot analysis using antibodies against FLAG (αFLAG) and GFP (αGFP) is shown.

To determine if dynamin 2 is indeed a stabilizer of the TLS, endogenous dynamin 2 in C2C12 cells was depleted by RNAi and its effect on TLS formation was analyzed. In control RNAi cells, numerous long BIN1-mediated TLS were formed and endogenous dynamin 2 was recruited to them (Fig. 2*A*, siCtrl) in the same manner as exogenously expressed human dynamin 2 (Fig. 1*B*). In contrast, formation of the TLS was strongly inhibited in dynamin 2 RNAi cells (Fig. 2*A*, siDnm2). Quantitative analyses showed that the relative amount of long TLS (≥5 μm) in dynamin 2 RNAi cells was significantly decreased (3.4 ± 0.4%) compared with that in control RNAi cells (10.3 ± 2.4%) (Fig. 2*B*). The efficiency of dynamin 2 depletion by RNAi was confirmed by immunoblot analysis (Fig. 2*C*). These results show that dynamin 2 is required for formation of stable TLS induced by BIN1 overexpression.

Previous studies demonstrated that PRD of dynamin 2 directly binds to SH3 domain of BIN1 (3). However, the contribution of their interaction in T-tubule biogenesis was unclear. To determine if the interaction between dynamin 2 and BIN1 is required for TLS formation, full length or a PRD truncated form of human dynamin 2 was exogenously expressed and their abilities to rescue dynamin 2 RNAi phenotypes were measured. Exogenously expressed FLAG-tagged human dynamin 2 completely rescued dynamin 2 RNAi phenotype in C2C12 cells with numerous TLS reconstituted to the same level as in control RNAi cells (11.5 ± 0.9%) (Fig. 2, *A*–*B*, siDnm2+DNM2-FLAG). In contrast, the PRD-truncated form of human dynamin 2 failed to rescue the membrane tubulation defects in dynamin 2 RNAi cells (3.4 ± 0.6%) (Fig. 2, *A*–*B*, siDnm2+DNM2ΔPRD-FLAG). Expression levels of exogenously expressed FLAG-tagged human dynamin 2 (wild-type and ΔPRD) in these experiments were confirmed by immunoblotting (Fig. 2*C*). Finally, we reconfirmed that full-length dynamin 2, but not PRD-truncated, human dynamin 2 binds to BIN1 by coimmunoprecipitation assay (Fig. 2*D*). These results suggest that interaction between dynamin 2 and BIN1 is required for formation of stable TLS.

**Figure 3:**
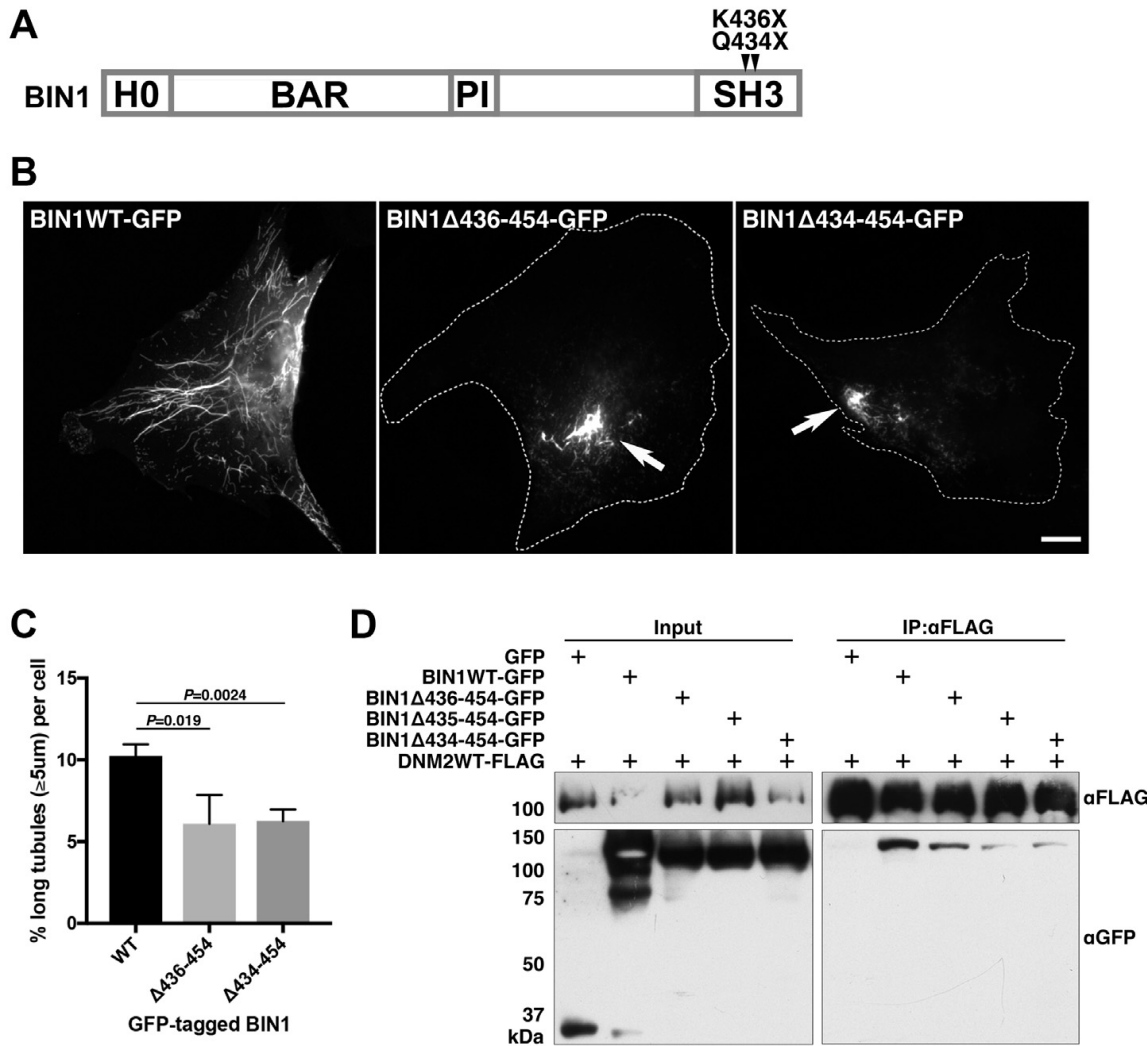
**Requirement of BIN1-dynamin 2 interaction in the TLS formation.***A*, schematic illustration of human BIN1 isoform 8. Two CNM mutations in SH3 domain, K436X and Q434X, are shown. *B*, defective TLS formation by BIN1 CNM mutants with partially truncated SH3 domain. Localization of GFP-tagged BIN1 of either wild-type (BIN1WT-GFP) or two CNM mutant (BIN1Δ436–454-GFP and BIN1Δ434–454-GFP). Abnormally aggregated TLS are indicated by arrows. Scale bar is 10 μm. *C*, quantitative analysis of long TLS (≥5 μm) formed by wild-type and mutant BIN1. Proportion of TLS formed by GFP-tagged wild-type BIN1 (WT) or by BIN1 mutants with partially truncated SH3 (Δ436–454 and Δ434–454) were 10.2 ± 0.7%, 6.1 ± 1.8%, and 6.3 ± 0.7%, respectively. Data are means ± SD (n > 600 TLS, N = 3). *D* reduced dynamin 2 interaction by mutant BIN1 with partially truncated SH3 domain. Coimmunoprecipitation analysis of wild-type dynamin 2 (DNM2WT-FLAG) with either wild-type (BIN1WT-GFP), mutant BIN1 with partially truncated SH3 domain (BIN1Δ436–454-GFP, BIN1Δ435–454-GFP, BIN1Δ434–454-GFP) or negative control (GFP). Immunoblots using anti-FLAG (αFLAG) and anti-GFP (αGFP) for input or immunoprecipitant using anti-FLAG (IP:αFLAG) are shown.

The protein encoded by an autosomal recessive CNM mutant of BIN1 harboring a nonsense mutations in the SH3 domain, p.K575∗ (p.K436∗ in isoform 8), had previously been shown to have reduced binding affinity with dynamin 2 (3). To examine if this alteration in the interaction between BIN1 and dynamin 2 is responsible for CNM pathogenesis, we examined TLS arising from the overexpression of CNM mutant BIN1 in SH3 domain (Fig. 3*A*). As described above, wild-type BIN1 induced numerous long TLS in C2C12 cells (Fig. 3*B*, BIN1WT-GFP). In contrast, BIN1 harboring the CNM mutations in SH3 domain, K436X and Q434X (dubbed BIN1Δ436–454 and BIN1Δ434–454, respectively), formed short and abnormally aggregated TLS (Fig. 3*B*, BIN1Δ436–454-GFP and BIN1Δ434–454-GFP). Quantitative analyses showed that the proportion of long TLS (≥5 μm) formed by wild-type BIN1 (10.2 ± 0.7%) was reduced when BIN1Δ436–454 or BIN1Δ434–454 was used (6.1% ± 1.8% and 6.3 ± 0.7%, respectively) (Fig. 3*C*). Consistent with the previous observations (3), CNM mutant BIN1 (BIN1Δ436–454 and BIN1Δ434–454), as well as BIN1 H435X (BIN1Δ435–454) where histidine at codon 435 was substituted with a premature termination codon, exhibited reduced binding affinity with dynamin 2 (Fig. 3*D*). Defective interaction of the CNM mutant BIN1 (BIN1Δ436–454 and BIN1Δ434–454) with dynamin 2 was also confirmed by reciprocal immunoprecipitation of GFP-tagged BIN1 using GFP-Trap (Fig. S2). These data suggest that physical interaction of dynamin 2 with BIN1 is required for formation of the TLS and their defective interaction is associated with CNM pathogenesis.

### Dynamin 2 CNM mutants form aggregates with defective membrane tubulation activity

**Figure 4:**
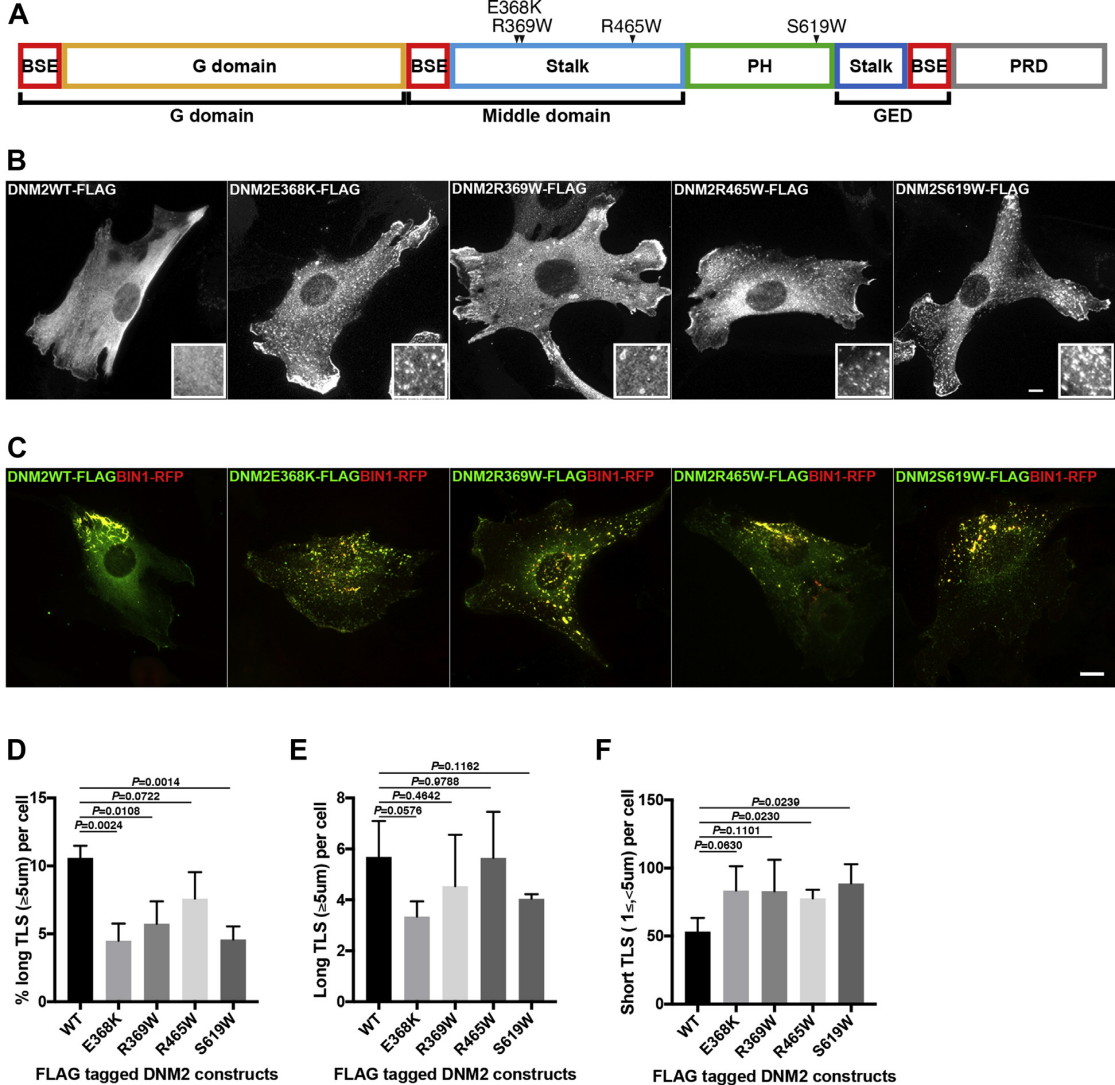
**CNM mutant dynamin 2 form aggregates with defective TLS formation.***A*, schematic illustrations of dynamin 2 variants used in this study. *B*, localization of FLAG-tagged dynamin 2 of either wild-type (DNM2WT-FLAG) or CNM mutants (DNM2E368K-FLAG, DNM2R369W-FLAG, DNM2R465W-FLAG, and DNM2S619W-FLAG). Scale bar is 10 μm. *C*, defective TLS formation in the presence of CNM mutant dynamin 2. Merged images of FLAG-tagged wild-type (DNM2WT-FLAG) or CNM mutant dynamin 2 (DNM2E368K-FLAG, DNM2R369W-FLAG, DNM2R465W-FLAG, and DNM2S619W-FLAG) (*green*) with BIN1-RFP (*red*) are shown. Scale bar is 10 μm. *D*, quantitative analyses of TLS formation. Proportion of the long TLS (≥5 μm) formed in the presence of mutant dynamin 2 were decreased (E368K: 4.5 ± 1.3%; R369W: 5.7 ± 1.7%; R465W: 7.6 ± 2.0%; S619W: 4.6 ± 1.0%) compared with that of wild-type dynamin 2 (10.6 ± 0.9%). Data are means ± SD (n > 500 TLS, N = 3). E, quantification of the number of long TLS (≥5 μm) per cell formed in the presence of wild-type or CNM mutant dynamin 2. The average number of long TLS per cell in the presence of wild-type dynamin 2 (5.7 ± 1.4) was slightly decreased by overexpressing mutant dynamin 2 (E368K:3.3 ± 0.6; R369W: 4.5 ± 2.0; R465W: 5.7 ± 1.8; S619W: 4.0 ± 0.2). Data are means ± SD (n ≥ 45 TLS, N = 3). *F*, quantification of the number of short TLS (1 μm ≤ and < 5 μm) per cell formed in the presence of wild-type or CNM mutant dynamin 2. The average number of short TLS per cell in the presence of wild-type dynamin 2 (53.2 ± 10.0) was significantly increased by overexpression of CNM mutant dynamin 2 (E368K: 83.4 ± 17.9; R369W: 82.9 ± 23.1; R465W: 77.7 ± 6.4; S619W: 88.6 ± 14.2). Data are means ± SD (n > 450, N = 3).

We next examined localization and function of four previously reported dynamin 2 CNM mutants (Fig. 4*A*) using the *in cellulo* TLS reconstitution assay. *DNM2*-related CNM patients typically present milder and slowly progressive symptoms and favorable prognosis, with an age of onset that varies from infantile to adolescence. Among the CNM mutations examined, E368K and S619W are known to be associated with more severe phenotypes (neonatal to childhood onset), whereas R369W and R465W are associated with milder symptoms and later onset (adolescence to adulthood) (30, 31). In C2C12 cells, FLAG-tagged wild-type dynamin 2 formed very fine puncta with some accumulation at the leading edge (Fig. 4*B*, DNM2WT-FLAG). In contrast, cells expressing mutant dynamin 2, with missense mutations either in the stalk (E368K, R369W and R465W) or in the PH domain (S619W), formed abnormally larger puncta with strong accumulation at the leading edges (Fig. 4*B*, DNM2E368K-FLAG, DNM2R369W-FLAG, DNM2R465W-FLAG, and DNM2S619W-FLAG). The effects of these mutant dynamin 2 forms on TLS formation were also examined. As described above, we observed wild-type dynamin 2 induced and was recruited to unevenly distributed membranous TLS (Fig. 4*C*, DNM2WT-FLAG). In contrast, mutant dynamin 2 induced shorter and more evenly distributed TLS despite still being colocalized with BIN1 (Fig. 4*C*, DNM2E368K-FLAG, DNM2R369W-FLAG, DNM2R465W-FLAG, and DNM2S619W-FLAG). Quantitative analyses showed that the proportion of the long TLS (≥5 μm) formed in the presence of mutant dynamin 2 was reduced (E368K: 4.5 ± 1.3%; R369W: 5.7 ± 1.7%; R465W: 7.6 ± 2.0%; S619W: 4.6 ± 1.0%) compared with that of wild-type dynamin 2 (10.6 ± 0.9%) (Fig. 4*D*). This is consistent with a possible overfission of the TLS. Indeed, overexpression of mutant dynamin 2 induced slightly decreased number of long TLS (≥5 μm) (Fig. 4*E*) and significantly increased number of short TLS (1 μm ≤ and < 5 μm) (Fig. 4*F*), although the statistical significance of this was modest. Taken together, these results suggest that mutant dynamin 2 variants exhibit enhanced membrane fission activities and different levels of aberrant dynamics that correlate with the severity of the symptoms in patients.

### CNM mutant dynamin 2 has constitutively active GTPase activity

**Figure 5:**
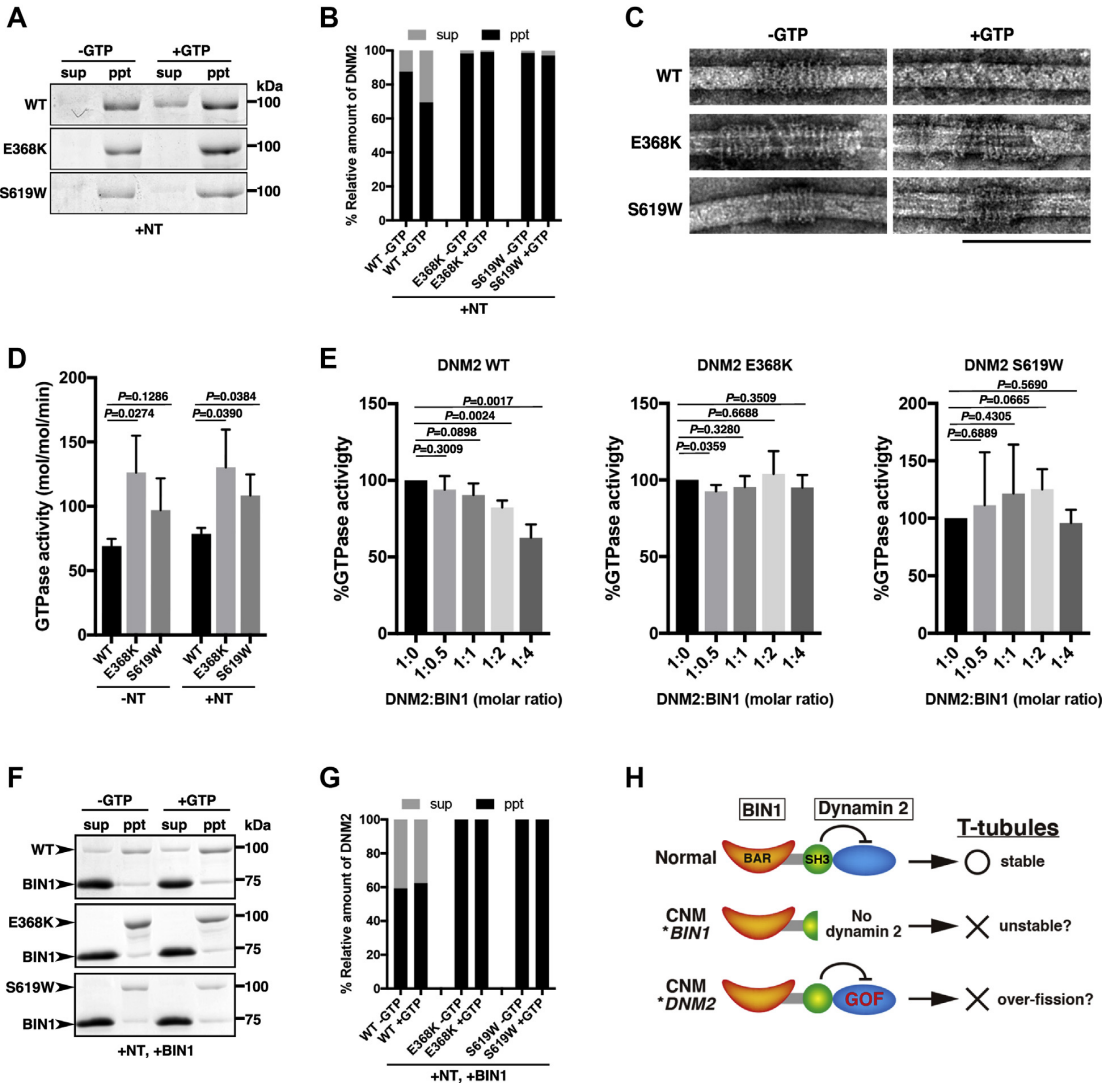
**Gain of function features of mutant dynamin 2 induce constitutive active GTPase activity.***A*, CNM mutant dynamin 2 form aggregates resistant to GTP hydrolysis. CBB-stained SDS-PAGE gel images of the *in vitro* sedimentation assay for either wild-type (WT) or mutant (E368K and S619W) dynamin 2 fractionated in the supernatant (sup) or in the precipitate (ppt) with or without GTP (+GTP or −GTP) in the presence of lipid nanotubes (+NT). *B*, quantification of the *in vitro* sedimentation assay. Relative amount of wild-type (WT) or mutant dynamin 2 (E368K and S619W) either in the supernatant (sup) or in the precipitate (ppt) with or without GTP (+GTP or −GTP) is shown. *C*, formation of GTP-resistant helical structures by mutant dynamin 2. Electron microscopic images of wild-type (WT) or mutant (E368K and S619W) dynamin 2 on lipid nanotubes in the absence (−GTP) or presence of GTP (+GTP). Scale bar is 200 nm. *D*, GTPase activity of wild-type (WT) and mutant (E368K and S619W) dynamin 2 in the absence (−NT) and presence (+NT) of lipid nanotubes. Data are means ± SD (n = 3, N = 3). *E*, insufficient suppression of GTPase activity of mutant dynamin 2 by BIN1. Relative GTPase activities of either wild-type (DNM2WT) or mutant dynamin 2 (DNM2E368K and DNM2S619W) with increasing amount of BIN1 (1:0, 1:0.5, 1:1, 1:2, and 1:4 in molar ratio) are shown. Data are means ± SD (n = 3, N = 3). *F*, BIN1 suppresses self-assembly of dynamin 2. CBB-stained SDS-PAGE gel images for wild-type (WT) or mutant (E368K and S619W) dynamin 2 fractionated in either the supernatant (sup) or in the precipitate (ppt) with or without GTP (+GTP or −GTP) in the presence of lipid nanotubes and BIN1. *G*, quantification of the *in vitro* sedimentation assay shown in (*F*). Relative amount of wild-type (WT) or mutant dynamin 2 (E368K and S619W) either in the supernatant (sup) or in the precipitate (ppt) with or without GTP (+GTP or −GTP) is shown. *H*, proposed model for defective T-tubule formation by CNM mutants of BIN1 and DNM2.

To further investigate the mechanisms of TLS formation by mutant dynamin 2, we examined a stalk domain mutant (E368K) and a PH domain mutant (S619W) for their self-assembly and GTPase activity, both of which are essential for membrane fission by dynamin (32, 33). Previous studies demonstrated that dynamin is self-assembled in a low salt condition and is disassembled in the presence of GTP (34, 35). Purified recombinant proteins of wild-type and E368K and S619W dynamin 2 mutants (Fig. S1*B*) self-assembled with lipid nanotubes with more than 85% precipitated before GTP addition (Fig. 5, *A*–*B*, −GTP). The wild-type dynamin 2 was disassembled after GTP addition and more than 30% of the proteins could be recovered in the supernatant (Fig. 5, *A*–*B*, WT, +GTP). In contrast, more than 95% of the mutant dynamin 2 remained in the precipitate even in the presence of GTP (Fig. 5, *A*–*B*, E368K and S619W, +GTP). To confirm that the precipitates of wild-type and mutant dynamin 2 were not nonspecific aggregates, we directly observed their structures using electron microscopy. Both wild-type and mutant dynamin 2 assembled into orderly helical structures on the lipid nanotube in the absence of GTP (Fig. 5*C*, −GTP). Although wild-type dynamin 2 was disassembled by GTP addition (Fig. 5*C*, WT, +GTP), mutant dynamin 2 remained in the helical structures even after 10 min incubation with 1 mM GTP (Fig. 5*C*, E368K and S619W, +GTP). These results suggest that CNM-associated mutant dynamin 2 proteins show enhanced self-assembly and form stable aggregates that resist GTP hydrolysis-dependent disassembly.

Previous studies showed that CNM mutant dynamin 2 has enhanced GTPase activity (28, 35, 36). Since self-assembly of dynamin stimulates its GTPase activity (32), we also speculated that the mutant dynamin 2 GOF phenotype arises *via* its GTPase activity. Indeed, both E368K and S619W demonstrated higher GTPase activities compared with that of wild-type irrespective of additional lipid nanotubes (Fig. 5*D*). Interestingly, purified recombinant BIN1 (Fig. S1B, BIN1 WT) stoichiometrically inhibited the GTPase activity of the wild-type dynamin 2 (Fig. 5*E*, DNM2WT), and this regulation similarly occurred with lipid nanotubes (Fig. S3, WT). In contrast, the BIN1-dependent regulation did not work efficiently on the mutant dynamin 2 and their GTPase activities remained high even in the presence of increasing levels of BIN1 (Fig. 5*E*, DNM2E368K and DNM2S619W) and were constitutively active even in the presence of lipid nanotubes (Fig. S3, E368K and S619W). Finally, we examined effects of BIN1 on self-assembly of dynamin 2. Self-assembly of wild-type dynamin 2 was suppressed in the presence of wild-type BIN1 protein and more than 40% could be recovered from the supernatant before GTP addition (Fig. 5, *F*–*G*, WT). In contrast, almost all mutant dynamin 2 remained in the precipitate even after 10 min incubation with 1 mM GTP in the presence of BIN1 (Fig. 5, *F*–*G*, E368K and S619W). These data suggest that BIN1 has a novel role in regulating the GTPase activity of dynamin 2 and that dysregulation arising in CNM patients with dynamin 2 mutations is due to constitutively active GTPase activity, enhanced membrane fission, and consequential myocyte dysfunction that is tightly linked to the pathogenesis of CNM.

## Discussion

In this study, we demonstrated using an *in cellulo* reconstitution assay that dynamin 2 has a novel role in stabilizing TLS (Figs. 1 and 2). Previous studies showed that dynamin 2 is closely localized at T-tubules in developing mouse skeletal muscle (26), suggesting a role in T-tubule biogenesis. Indeed, muscle biopsies from CNM patients with mutations in dynamin 2 (p.E368K and p.R465W) showed disorganization of T-tubules and triad structures (17). Furthermore, ectopic expression of CNM mutant dynamin 2 in adult mouse (23), *Drosophila* (28), and zebrafish (37) induced abnormally shaped or fragmented T-tubules. These studies did not elucidate a possible function for dynamin 2 in T-tubule biogenesis; however, our *in cellulo* reconstitution assay data suggest that dynamin 2 is required for the correct formation and/or maintenance of T-tubules.

The PRD of dynamin 2 was found to be indispensable for stable formation of BIN1-induced TLS (Fig. 2). Consistently, CNM mutant BIN1 with partially truncated SH3 domain showed membrane tubulation defects and reduced binding affinity with dynamin 2 (Fig. 3). To date, various CNM-related *BIN1* mutations had been identified in H0 domain (p.V18E, p.K21Δ, p.R24C, p.K35N, and p.A36E), BAR domain (p.R145C, p.D151N, p.R154Q, and p.R234C), PI domain (c.IVS10-1G > A), and SH3 domain (p.Q573∗, p.K575∗, P593HfsX54, X594DfsX53) (3, 4, 5, 6, 7, 8, 9, 10, 17). Among them, mutations in H0, BAR, and PI caused membrane tubulation defects both *in vitro* and *in vivo*, and disorganized T-tubule formation (3, 4, 5, 7, 8, 9). In contrast, pathogenic mechanisms caused by mutations in SH3 domain of BIN1 were poorly understood except for the fact that K575X mutant showed less binding affinity with dynamin 2 (3). Previous studies in mice showed that T-tubules are initially formed in longitudinal orientation and then reoriented to form their eventual transverse orientation during muscle development (38). Interestingly, dynamin 2 colocalized with BIN1 only on the longitudinal T-tubules in early development and is only observed at the Z-line of sarcomeres in adult mice (26). Thus, it is possible that BIN1 interacts with dynamin 2 only at specific developmental stages of myofiber formation. We also showed that SH3 domain mutant BIN1 protein formed aggregated TLS (Fig. 3*B*). Skeletal muscle biopsies from CNM patients with p.Q573∗ (corresponding to p.Q434∗ in isoform 8) show perinuclear aggregation and enlarged T-tubules (17). Thus, alternative pathogenic mechanisms by SH3 domain mutant BIN1 may be due to formation of malfunctioning “abnormally aggregated” T-tubules during skeletal muscle development.

Previous studies showed that GTP hydrolysis is crucial for membrane fission by dynamin (18, 19, 39). In this process, dynamin is self-assembled to form helical structures so that the G domains of dynamins from neighboring turns interact with each other. These interactions activate the GTPase activity of dynamin and subsequent structural changes of helical structures upon GTP hydrolysis lead to membrane fission (40). Consistent with previous studies (28, 35, 36), we demonstrated that CNM-related mutant dynamin 2 forms abnormal aggregates (Fig. 4*B*) and shows enhanced GTPase activities (Fig. 5). Since mutant dynamin 2 remained in the orderly helical structures even after GTP hydrolysis (Fig. 5*C*), it is possible that continual contacts of multiple dynamin 2 molecules *via* their G-domains maintain them in a constitutively active GTPase state, thus causing excessive fission of the T-tubules *in vivo*.

In this study, we have analyzed previously reported dynamin 2 variants identified from CNM patients in an *in cellulo* assay. All the dynamin 2 variants formed abnormal aggregates inducing shorter TLS (Fig. 4). This phenotype suggests that these *DNM2*-associated CNM mutations are responsible for a GOF of the encoded proteins in both self-assembly and GTPase activity. Indeed, mutant dynamin 2 (E368K and S619W) self-assembled *in vitro* to form GTP-resistant aggregates with elevated GTPase activity (Fig. 5). Among these dynamin 2 mutants examined, R465W showed the mildest phenotypes both in disease symptoms (30, 31) and in TLS formation (Fig. 4) in comparison with other mutant dynamin 2. Interestingly, E368 and R369 alterations in the stalk (middle domain) and S619 alteration in the PH domain colocated in close 3D proximity at the PH-stalk interface based on published structure of dynamin 1 monomer (30, 31). In contrast, R465W is altered in a helical structure distal to the stalk–PH interface (30, 31). Previous structural analysis of dynamin 1 and 3 showed that the PH domain of dynamin is flipped back to interact with the stalk and GTPase domain to form an inhibitory “closed” state, and this state is released upon binding of the PH domain with membrane (21, 41). Thus, it is possible that the CNM mutations we investigated affect the structural configuration of PH and stalk domains to alter regulation of the GTPase activity into a constitutively active GTPase state. In contrast, dynamin 2 with the R465W mutation causes structural alteration to the stalk domain self-assembly leading to elevated GTPase activity. Future structural studies of these mutant dynamin 2 variants will be needed to explain the precise mechanisms of their GOF phenotype linked to the pathogenesis of CNM.

BIN1 has been reported to negatively regulate the GTPase activity of dynamin 2 in the presence of lipids (26). In contrast, we consistently demonstrated that the GTPase activity of dynamin 2 was stoichiometrically inhibited by BIN1 regardless of the presence of lipids (Fig. 5*E* and Fig. S3). Interestingly, GTPase activity of mutant dynamin 2 is insufficiently inhibited by BIN1 and its GTPase activities remained active even in the presence of BIN1 (Fig. 5*E* and Fig. S3). Our previous studies showed that amphiphysin 1, a neuronal BIN1 isoform, regulates the GTPase activity of dynamin 1, the neuronal dynamin isoform, in a stoichiometry-dependent manner (42, 43). Another BAR domain protein endophilin also interacts with dynamin 1 and inhibits its GTPase activity (44). BIN1 is increasingly expressed as skeletal muscle development progresses, while expression level of dynamin 2 remains unchanged (14). Thus, it is tempting to speculate that BIN1 contributes not only to membrane tubulation *per se*, but it also supports membrane stabilizing function of dynamin 2 by suppressing its GTPase activity to establish T-tubule system during normal development (Fig. 5*H*, Normal). In contrast, regulation of membrane remodeling by the BIN1-dynamin 2 complex may be compromised in CNM patients by causing unstable T-tubules in *BIN1*-associated CNM (Fig. 5*H*, CNM ∗*BIN1*) or overfission of T-tubules in *DNM2*-associated CNM patients (Fig. 5*H*, CNM ∗*DNM2*).

In this study, using *in cellulo* assay, we found that the aberrant membrane remodeling by mutated BIN1 and dynamin 2 is tightly correlated with the pathogenesis of CNM in patients harboring *BIN1* or *DNM2* gene alterations. Recent development of next-generation sequencing technologies provides us with enormous amount of genomic data from patients with various genetic diseases; however, it is crucial to discriminate disease-causing mutations from neutral variants. Our *in cellulo* approaches to analyze myocyte defects should help to provide both mechanistic insights and increased precision in the diagnosis of CNM and related inherited disease.

## Experimental procedures

### Molecular biology

Expression constructs used in this study were generated using Gateway Cloning Technology (Thermo Fisher Scientific). Entry clones of human dynamin 2 (NM_001005360) and human BIN1 isoform 8 (NM_004305.4) were prepared by B-P recombination cloning into pDONR201 vector of PCR products respectively amplified from pcDNA3.1-GFP-Topo-hDNM2-WT (generous gift from P. Guicheney, UPMC) and pEGFP-mAmph2 (generous gift from P. De Camilli, Yale University) using corresponding primers detailed in Table S1. Expression constructs of dynamin 2 and BIN1 were prepared by L-R recombination cloning of their Entry clones into Destination vectors either for expressing proteins in mammalian cells (pCI vectors for expressing FLAG-, GFP-, or RFP-tagged proteins) or for bacterial protein expression (pET15b for His-fusions and pGEX-6P-2 for GST-fusions) (generous gift from H. McMahon, MRC-LMB).

### Cell culture, DNA transfection, and RNAi

C2C12 cells (ATCC CRL-1722) and HEK293T cells (ATCC CRL-3216) were grown in D-MEM (High Glucose) with L-Glutamine, Phenol Red, and Sodium Pyruvate (043-30085, FUJIFILM Wako chemicals) supplemented with 10% fetal bovine serum (FBS) (12483020, Thermo Fisher Scientific) and Penicillin-Streptomycin (100 unit/ml) (15140122, Thermo Fisher Scientific) at 37 °C in 5% CO_2_. For transfection of C2C12, 70% confluent cells in VIOLAMO VTC-P24 24-well plates (2-8588-03, AS ONE) were transfected with 0.5 μg expression plasmids using Lipofectamine LTX with Plus Reagent (15338100, Thermo Fisher Scientific). To examine consequences of the expression of BIN1 or dynamin 2 in either wild-type or mutant forms, cells were fixed after 48 h of the transfection for phenotypic analyses. To transfect HEK293T, 70% confluent cells in VIOLAMO VTC-P6 6-well plates (2-8588-01, AS ONE) were transfected with 1.5 μg expression plasmids with Lipofectamine LTX with Plus Reagent. The cells were collected 48 h after the transfection and used for coimmunoprecipitation analysis. For RNAi treatment of C2C12 cells, 70% confluent cells in 24-well plates were transfected with 10 pmol of either siGENOME Mouse Dnm2 (13430) siRNA–SMARTpool (M-044919-01, Dharmacon) or siGENOME nontargeting siRNA Pools #1 (D-001206-13-05, Dharmacon) with Lipofectamine RNAiMAX Transfection Reagent (13778150, Thermo Fisher Scientific). For the rescue experiments, cells were also transfected with corresponding expression plasmids at 24 h after the RNAi started. To examine the consequences of the RNAi, cells were collected at 72 h after the RNAi started and used for phenotypic or immunoblot analyses.

### Antibodies

Primary antibodies used in this study were polyclonal rabbit anti-DDDDK tag (MBL, PM020), polyclonal goat anti-Dynamin2 (C-18) (sc-6400, Santa Cruz Biotechnology), monoclonal mouse anti β-actin (A5441, Merck), and monoclonal rabbit anti-GFP (D5.1) XP (2956, CST). All the secondly antibodies used in this study, Alexa Fluor 488-conjugated donkey anti-rabbit IgG (H + L) (A21206), Alexa Fluor 555-conjugated donkey anti-rabbit IgG (H + L) (A31572), Alexa Fluor 568-conjugated donkey antigoat IgG (H + L) (A11057), HRP-conjugated rabbit antigoat IgG (H + L) (31402), HRP-conjugated rabbit antimouse IgG (H + L) (31450), HRP-conjugated goat anti-rabbit IgG (H + L) (31460) were purchased from Thermo Fisher Scientific.

### Immunostaining of C2C12 cells

For immunostaining of C2C12, cells grown on coverslips were fixed with 4% paraformaldehyde (PFA) diluted in PBS from 16% PFA solution (15710, Electron Microscopy Sciences) for 15 min at room temperature. After washing with PBSTB (PBS containing 0.1% Triton X-100, 1% BSA), the cells were permeabilized and blocked with PBS containing 0.5% Triton X-100 and 3% BSA for 1 h at room temperature. The samples were then incubated with primary antibodies diluted 1:1000 in PBSTB overnight at 4 °C in a humid chamber. After washing with PBSTB, the cells were incubated with secondly antibodies diluted in PBSTB for 3 h at room temperature. Then, the cells were washed with PBSTB and mounted in Fluoromount/Plus (K048, Diagnostic BioSystems).

### DiO staining of C2C12 cells

DiO (DiOC18(3) (3,3′-Dioctadecyloxacarbocyanine Perchlorate) (D275, Molecular Probes) was dissolved in 5 mM in DMSO and stocked at −30 °C. C2C12 cells expressing BIN1-RFP were incubated in 5 μM DiO in cell culture medium for 20 min at 37 °C. After three times washing with PBS, the cells were fixed with 4％ PFA in PBS and used for microscopy.

### Microscopy

Fixed C2C12 cells were visualized using BX51 fluorescence microscope (OLYMPUS) with 40 × NA 0.75 objective lens and images were acquired with Discovery MH15 CMOS camera (Tucsen) and ISCapture image acquisition software (Tucsen). For live cell imaging of C2C12 cells expressing BIN1-GFP and dynamin 2-RFP, cells on 35 mm Glass Base Dish (3911-035, IWAKI) were maintained in 5% CO_2_ at 37 °C with a thermo-control system (MI-IBC, OLYMPUS) and images were acquired on IX71 microscope (OLYMPUS) fitted with X-Light spinning disc confocal unit (CrestOptics) and iXon EMCCD camera (DU-888E-C00-#BV, ANDOR) using MetaMorph (Molecular Devices). Three optical sections were captured at 15 s intervals with a 100 × NA 1.35 Oil Iris objective lens and a 2 × 2 bin. All images were analyzed using Fiji (45) and processed with Adobe Photoshop 2020 (Adobe).

### Introduction of CNM mutations into BIN1 and dynamin 2

Entry clones of mutant BIN1 in SH3 domain (BIN1Δ434–454, BIN1Δ435–454 and BIN1Δ436–454) were prepared by B-P recombination reaction of PCR products amplified from pEGFP-mAmph2 using corresponding primers (Table S1) into pDONR201. Entry clones for the CNM mutant of dynamin-2, R369W and R465W, were prepared by BP recombination reaction of PCR products amplified from pcDNA3.1-GFP-Topo-hDNM2-R369W and pcDNA3.1-GFP-Topo-hDNM2-R465W (generous gift from P. Guicheney, UPMC) using corresponding primers (Table S1) into pDONR201. Entry clones for other mutant dynamin 2 were prepared by introducing corresponding mutations into the Entry clone of wild-type human dynamin 2 using QuikChange Lightning site-directed mutagenesis kit (210518, Agilent) following manufacturer’s instruction. Sense and antisense primers used for the site-directed mutagenesis are shown in Table S1.

### Quantitative analysis of in cellulo membrane tubulation

Quantification of the BIN1-mediated TLS was performed by Fiji (45). Firstly, background signal was subtracted from microscopic images of BIN1-expressing cells (Rolling ball radius = 10 pixels). Then, the TLS were enhanced with FFT Bandpass Filter (Filter: large structures down to five pixels and up to three pixels; Suppress stripes: None; Tolerance of direction: 5%). The TLS were detected and binarized with threshold command and the binarized membrane tubules were skeletonized for analysis with Analyze Skeleton (2D/3D) plugin. TLS longer than or equal to 5 μm were considered as “long,” whereas those with length between 1 and 5 μm were considered as “short” based on the value of the Maximum Branch Length in the Analyze Skeleton.

### Immunoblot analysis

Cells were lysed and heat-denatured with SDS gel loading buffer for 5 min at 95 °C. The denatured proteins were separated on 10% polyacrylamide gel by SDS-PAGE using Mini-PROTEAN 3 system (Bio-Rad, 165-3301). The proteins were then transferred onto Amersham Protran Premium NC 0.45 (10600003, Cytiva) using Mini Trans-Blot Electrophoretic Transfer Cell (170-3930, Bio-Rad). The blot was blocked with blocking buffer (PBS containing 3% Skim Milk and 0.05% Tween 20) for 1 h at room temperature followed by 1 h incubation at room temperature with primary antibodies diluted 1:1000 in the blocking buffer. After washing, the blot was incubated with secondary antibodies diluted 1:10,000 in the blocking buffer for 1 h at room temperature. After washing with the blocking buffer, the blot was incubated with ECL Prime Western Blotting Detection Reagent (RPN2232, Cytiva) and the signal was detected using Hyperfilm ECL (28906836, Cytiva).

### Coimmunoprecipitation assay

For coimmunoprecipitation assay of BIN1 and dynamin 2, HEK293T cells transfected with their expression constructs were harvested in 400 μl of Extraction/Wash buffer (20 mM HEPES, 150 mM NaCl, 1 mM EDTA, 0.2% Triton X-100, 0.1 mM PMSF and complete proteinase inhibitor (11697498001, Merck), pH 7.4) and lysed with TAITEC VP-5S sonicator (output: 4; 5 s×3 times). For 350 μl of the cleared lysate obtained by centrifugation (20,600*g* for 10 min at 4 °C), 10 μl of Anti-DDDDK-tag mAb-Magnetic Agarose (M185-10, MBL) was added and FLAG-tagged protein was immunoprecipitated for 1 h at 4 °C with gentle agitation. For immunoprecipitation of GFP-tagged protein, GFP-Trap Magnetic Agarose (gtma, Chromotek) was alternatively used for the immunoprecipitation. After washing with Extraction/Wash buffer, proteins bound to the magnetic beads were eluted with SDS gel loading buffer and used for immunoblot analysis.

### Purification of recombinant BIN1 and dynamin 2

Recombinant protein of BIN1 isoform 8 was expressed and purified as GST fusion using Glutathione Sepharose 4B (17075601, Cytiva) according to the manufacturer’s instructions. GST-tag was removed by PreScission Protease (27084301, Cytiva), and purified BIN1 was recovered using Ultrafree-MC-GV Centrifugal Filters (UFC30GV00, Merck). Purified protein was concentrated to 2 mg/ml with centrifugal filter (Amicon Ultra-4, 10K, Merck) and stored at −80 °C. Wild-type and mutant dynamin 2 were expressed and purified as His-tagged proteins according to the purification protocol for dynamin1 and dynamin3 described previously (21) with some modifications. Rosetta2 (DE3) pLysS competent cells (71401, Merck) transformed with dynamin 2 cloned in pET23b were grown to 0.5 to 2 of OD 600 in 1 L LB medium, and protein expression was induced by adding 0.5 mM IPTG for 12 h at 18 °C. The cells were harvested by centrifugation at 5500*g* for 10 min using KUBOTA 7780 (AG-5006 rotor, KUBOTA). The cell pellet was resuspended by 10 ml PBS, and they were harvested again by centrifugation at 4500*g* for 20 min at 4 °C using KUBOTA 5900 (RS-480M rotor, KUBOTA). The cells were resuspended by Wash buffer (25 mM Hepes/NaOH, 500 mM NaCl, 2 mM MgCl_2_, 20 mM Imidazole, pH 7.8) containing 0.1 mg/ml DNase I (10104159001, Merck) and 0.1 mM Pefabloc SC (11429868001, Merck) and sonicated using Digital Sonifier 250D-Advanced (BRANSON) with the following condition (Pulse: ON 5.0 s, OFF 15.0 s, Time: 1 min 15 s and Amplitude: 50%). The cleared lysate was prepared by centrifugation at 32,800*g* for 30 min at 4 °C using KUBOTA 7780 (AG-508R rotor, KUBOTA), and it was treated with 50 μl/100 ml Benzonase (70664-3CN, Merck) for 30 min at 4 °C. His-tagged dynamin 2 in the cleared lysate was recovered by 600 l in bed volume of TALON Metal Affinity Resin (635502, Takara Bio) with gentle agitation for 1 h at 4 °C. After five times washing with the Wash buffer, the beads-bound dynamin 2 was eluted with Elution buffer (25 mM Hepes/NaOH, 500 mM NaCl, 2 mM MgCl_2_, 500 mM Imidazole, pH 7.8). Purified dynamin 2 in the eluates was concentrated with Amicon Ultra Centrifugal Filters (UFC205024, Merck) to 2 mg/ml in the stock buffer (25 mM Hepes/NaOH, 300 mM NaCl, 2 mM MgCl_2_, 1 mM EDTA, 1 mM EGTA and 1 mM DTT, pH 7.8) and stored at −80 °C.

### Preparation of LUVs and lipid nanotubes

Large unilamellar vesicles (LUVs) were prepared as previously described (46). For LUVs, 70% PS (840032C, Avanti), 10% biotinPE (870285X, Avanti), and 20% cholesterol (700000, Avanti) (w/v), and for lipid nanotubes, 40% NFA Galactocerebrosides (Sigma C1516), 40% PC (840051C, Avanti), 10% PI(4,5)P_2_ (524644, Calbiochem), and 10% cholesterol (700000, Avanti) (w/v) were mixed and dissolved in 250 μl of chloroform and 75 μl methanol in Mighty Vial No.01 4 ml (5-115-03, Maruemu). Then the solvent was evaporated using slow-flow nitrogen gas to produce a lipid film on the glass and then completely dried in a vacuum desiccator for 1 h. The dried lipid was rehydrated by water-saturated nitrogen gas followed by addition of 250 μl of filtered 0.3 M sucrose for 2 h at 37 °C. The resultant LUVs and lipid nanotubes were passed through 0.4 μm- and 0.2 μm-polycarbonate filters, respectively, 11 times using Avanti Mini extruder (Merck). The LUVs and lipid nanotubes (1 mg/ml of final concentration) were stored at 4 °C in dark to avoid photooxidation.

### EM imaging of *in vitro* assay

LUVs were diluted to 0.17 mg/ml in cytosolic buffer (25 mM Hepes, 25 mM KCl, 2.5 mM Magnesium acetate, 0.1 M K-glutamate, pH 7.2). Dynamin 2 and BIN1 were diluted to 2.3 μM in the cytosolic buffer. Formvar filmed EM grids were carbon-coated, then glow-discharged. Droplets of the diluted lipids (10 μl each) were prepared on Parafilm and adsorbed on EM grids for 5 min at room temperature. Then the EM grids with lipids were transferred to other droplets of the diluted dynamin 2-BIN1 complexes and incubated for 30 min at room temperature in a humid chamber. The EM grids were negatively stained with filtered 2% uranyl acetate and observed with transmission electron microscope (H-7650, HITACHI) at Central Research Laboratory in Okayama University Medical School.

### *In vitro* sedimentation assay

*In vitro* sedimentation assay of dynamin 2 was performed as described previously (35). In short, wild-type or CNM mutant (E368K and S619W) dynamin 2 was diluted to 1 μM in reaction buffer (10 mM Hepes, 2 mM MgCl_2_, 100 mM NaCl, pH 7.5) and incubated for 5 min at 37 °C in the presence of lipid nanotubes (0.01 μg/μl). To induce disassembly, 1 mM GTP was added to the preassembled dynamin 2 and incubated for 5 min at 37 °C. The samples were centrifuged at 230,000*g* for 10 min at 25 °C using CS100GXL ultracentrifuge and S120AT3 rotor (Eppendorf Himac Technologies), and resultant supernatant and pellet were analyzed by SDS-PAGE followed by Coomassie Brilliant Blue R-250 staining. To assess the effect of BIN1 on self-assembly and disassembly of dynamin 2, 4 μM of BIN1 was included in the same assay.

### Dynamin GTPase activity

GTPase activity of dynamin 2 was determined by monitoring release of free orthophosphate using malachite green assay (47). The malachite green reagent was prepared by mixing solution A (17 mg of Malachite Green Carbinol base dye (229105, Merck) in 20 ml 1 N HCl) and Solution B (0.5 g Ammonium molybdate (277908, Merck) in 7 ml 4 N HCl) with filling up to 50 ml by MilliQ water followed by filtration through 0.45 μm membrane (S-2504, KURABO). In the assay, 0.2 μM dynamin in the presence of BIN1 at different molar ratio was mixed with 1 mM GTP in GTPase reaction buffer (10 mM Hepes, 2 mM MgCl_2_, 50 mM NaCl, pH 7.5) with or without 0.005 μg/μl lipid nanotubes and incubated for 5 min at 37 °C. After the reaction was stopped on ice for 10 min, 160 μl of malachite green reagent was added to the 40 μl of the reaction mix in 96-well plate (442404, Thermo Fisher Scientific). After 5 min shaking at 1200 rpm with Digital MicroPlate Genie Pulse (Scientific Industries, Inc), released orthophosphate was colorimetrically quantified by measuring OD 650 nm using a microplate reader (SH-1000, CORONA ELECTRIC).

### Statistical data analysis

Statistical data analysis was performed using Prism 7 (GraphPad Software) and Excel (Microsoft). For all quantification provided, the means and SD are shown. Statistical significance was determined using a two-sided *t*-test and *p* values are shown in the figures.

## Data availability

All data are contained within the manuscript.

## Conflict of interest

The authors declare that they have no conflicts of interest with the contents of this article.
