# Supplementary material for: Mutant BIN1-Dynamin 2 complexes dysregulate membrane remodeling in the pathogenesis of centronuclear myopathy
Source: J Biol Chem. 2020 Nov 21;296:100077. doi: 10.1074/jbc.RA120.015184 (PMC7949082; doi:10.1074/jbc.RA120.015184)

SUPPORTING INFORMATION

Dysregulated membrane remodeling by mutant BIN1-Dynamin 2 complex in pathogenesis of centronuclear myopathy

Kenshiro Fujise[1], Mariko Okubo[2,3], Tadashi Abe[1], Hiroshi Yamada[1], Ichizo Nishino[2], Satoru Noguchi[2], Kohji Takei[1]* and Tetsuya Takeda[1]*

Table S1

**Wild type and mutant BIN1**	
BIN1 fw:	5'-ggggacaagtttgtacaaaaaagcaggctgcatggcagagatgggcag-3'
BIN1 wt rv:	5'-ggggaccactttgtacaagaaagctgggtctgggaccctctcagtgaag-3'
BIN1Δ434-454 rv:	5'-ggggaccactttgtacaagaaagctgggtcgttccagtcgctctccttc-3'
BIN1Δ435-454 rv:	5'-ggggaccactttgtacaagaaagctgggtctactggttccagtcgctct-3'
BIN1Δ436-454 rv:	5'-ggggaccactttgtacaagaaagctgggtcgtgctggttccagtcgc-3'
**Wild type and mutant (R369W and R465W) dynamin 2**	
Dynamin2 fw:	5'-ggggacaagtttgtacaaaaaagcaggctgcatgggcaaccgcggga-3'
Dynamin2 rv:	5'-ggggaccactttgtacaagaaagctgggtcgtcgagcagggatggc-3'
**Other mutant dynamin2 (for site directed mutagenesis)**	
Dynamin2 E368K sense:	5'-tcaatcgcatcttccacaagcggttcccatttgag-3'
Dynamin2 E368K antisense:	5'-ctcaaatgggaaccgcttgtggaagatgcgattga-3'
Dynamin2 S619W sense:	5'-gctggaaggcctggttcctccgagc-3'
Dynamin2 S619W antisense:	5'-gctcggaggaaccaggccttccagc-3'

Figure S1 In cellulo and in vitro membrane tubulation by BIN1 and dynamin 2.
(a) TLS is a membranous structure. Fluorescent microscopic images of C2C12 cells overexpressing RFP-tagged BIN1 counter-stained with a membrane dye DiO. BIN1 (BIN1-RFP) and DiO are shown in red and green in the merged image. Scale bar is 10 μm. (b) Purification of recombinant human BIN1 and dynamin 2. CBB stained gel images after SDS-PAGE for purified BIN1 (BIN1 WT) and wild type (Dynamin 2 WT) or CNM mutant (Dynamin 2 E368K and S619W) dynamin 2. Arrowheads indicate the corresponding bands for the purified proteins. (c) TEM images of *in vitro* reconstituted membrane tubules induced by BIN1 alone (BIN1) or BIN1 with wild type dynamin 2 (BIN1+DNM2). Scale bar is 200 nm.

Figure S2 Defective interaction of CNM mutant BIN1 with dynamin 2.
Co-immunoprecipitation of GFP-tagged wild type BIN1 (BIN1 WT-GFP) or CNM mutant BIN1 (BIN1Δ436-454-GFP and BIN1Δ434-454-GFP) with FLAG-tagged wild type dynamin 2 (Dynamin 2 WT-FLAG). Immunoblot analysis using antibodies against FLAG (α-FLAG) and GFP (α-GFP) for input and co-IP samples with GFPtrap are shown. Quantitative analysis shows stoichiometry of wild type BIN1 with dynamin 2 is 1:0.3, while those for CNM mutant BIN1 are 1:0.

Figure S3 Inefficient BIN1-mediated regulation of GTPase activity of CNM mutant dynamin 2
GTPase activity of wild type (WT) or CNM mutant (E368K and S619W) dynamin 2 in the absence (-BIN1) or presence of BIN1 in 1:4 molar ratio (+BIN1). GTPase activities (mol/mol/min) for wild type or mutant dynamin 2 (E368K and S619W) are 69.7 ± 2.3, 116.3 ± 10.2 and 103.5 ± 7.4 in the absence of BIN1 and 44.7 ± 6.6, 111.8 ± 10.2 and 127.1 ± 19.1, respectively. Data are means ± SD (n=3, N=3).

Movie S1. BIN1-GFP in C2C12 cells.

Movie S2. BIN1-GFP in the presence of dynamin 2-RFP in C2C12 cells.

Figure S1 Fujise et al

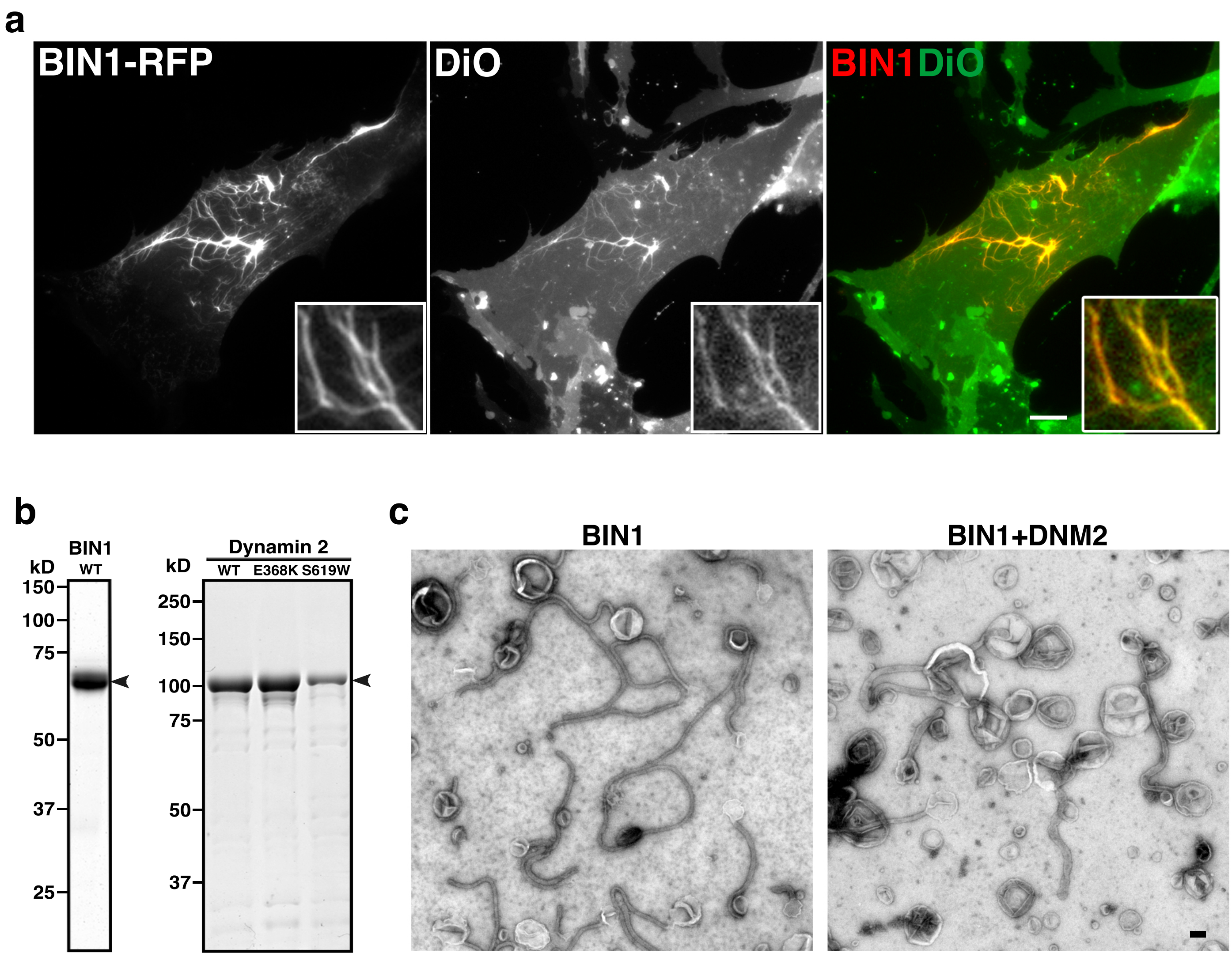

Figure S2 Fujise et al

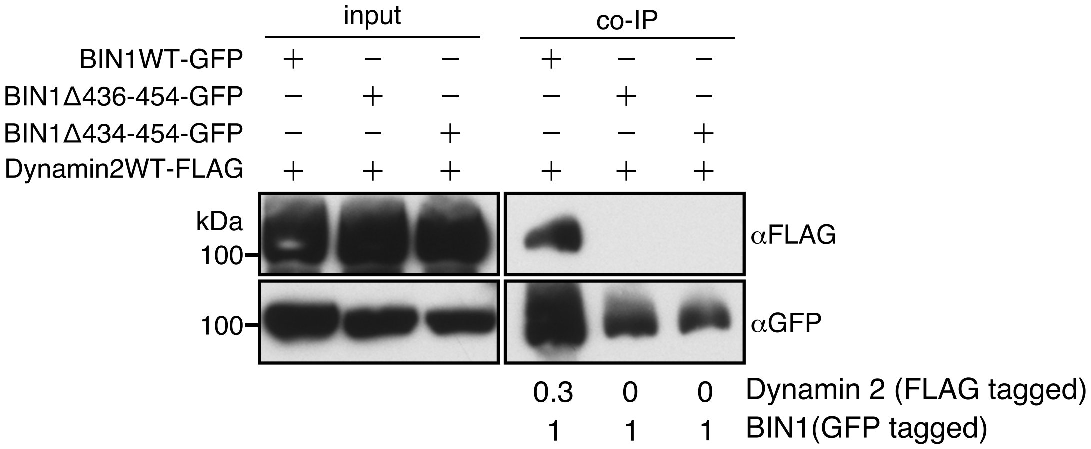

Figure S3 Fujise et al

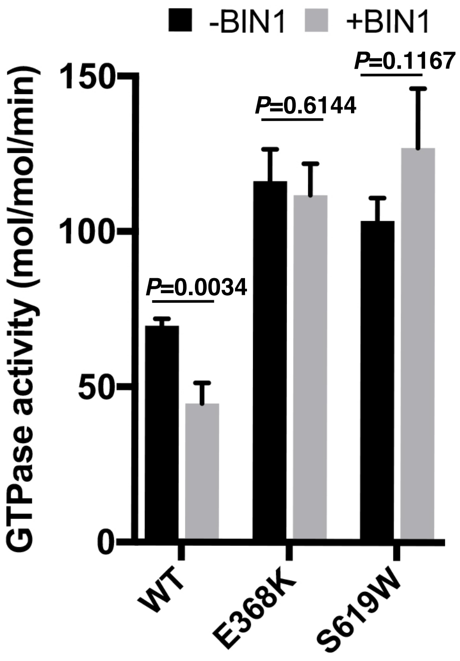

Supplement: Supplementary Figures and Table [file mmc1.pdf]
